# Late-onset ornithine transcarbamylase deficiency caused by a somatic mosaic mutation

**DOI:** 10.1038/s41439-018-0022-x

**Published:** 2018-08-16

**Authors:** Tomoko Lee, Maiko Misaki, Hideki Shimomura, Yasuhiko Tanaka, Satoru Yoshida, Kei Murayama, Kimitoshi Nakamura, Ryoji Fujiki, Osamu Ohara, Hideo Sasai, Toshiyuki Fukao, Yasuhiro Takeshima

**Affiliations:** 1 Department of Pediatrics, Hyogo College of Medicine, Nishinomiya, Japan; 2 Department of Pediatrics, Seirei Mikatahara General Hospital, Hamamatsu, Japan; 3 Center for Medical Genetics and Department of Metabolism, Chiba Children’s Hospital, Chiba, Japan; 4 Department of Pediatrics, Kumamoto University Graduate School of Medicine, Kumamoto, Japan; 5 Department of Technology Development, Kazusa DNA Research Institute, Chiba, Japan; 6 Department of Pediatrics, Gifu University Graduate School of Medicine, Gifu, Japan

## Abstract

An 18-month-old boy was diagnosed with late-onset ornithine transcarbamylase deficiency. Genetic analysis revealed a mosaic frameshift mutation (p.Q279fs) in the *OTC* gene. Despite the presence of a null mutation, he exhibited a milder phenotype, suggesting that the wild-type allele could rescue the function of OTC. The presence of mosaicism has great effects on the clinical phenotype and recurrence-risk assessment, which should be taken into consideration for genetic counseling.

Ornithine transcarbamylase (OTC) deficiency (OTCD) (MIM#311250) is the most common urea cycle disorder caused by mutations in the *OTC* gene located on chromosome Xp21^1,2^. OTCD causes hyperammonemia, presenting with clinical manifestations such as lethargy, vomiting, coma, and in severe cases, death^3^. Most of the OTCD patients are hemizygous males, and approximately 20% of the female carriers of *OTC* mutations are also symptomatic^4–6^.

OTCD is classified into two groups: severe neonatal-onset and late-onset phenotypes. Neonatal-onset OTCD presents with acute hyperammonemia within the first few days of life, whereas late-onset OTCD can present later from infancy to adulthood^7^. Genotype−phenotype correlations have been well defined. Null mutations, such as large deletions, frameshift, and nonsense mutations, and some missense mutations resulting in a complete loss of OTC function cause severe neonatal-onset diseases in hemizygous males and in most symptomatic heterozygous females^3,8–10^. Missense mutations that retain some OTC activity cause late-onset disease in hemizygous males^3,8–10^.

Mosaicism is a well-established biological phenomenon. The frequency of mosaicism appears to vary among different disease genes. To date, somatic mosaicism in the *OTC* gene has been reported in only a few cases^11–14^.

Here, we investigate late-onset OTCD in a boy caused by a somatic mosaic frameshift mutation.

An 18-month-old Japanese boy born to nonconsanguineous parents as the third child with a healthy elder sister and a brother was referred to the hospital due to vomiting and unconsciousness. His mental and physical development was normal to date. Blood examination revealed elevated ammonia levels (435 µg/dl, normal range: 30–70) and glutamine (1011.5 nmol/ml, normal range: 422.1–703.8), whereas citrulline (7.4 nmol/ml, normal range: 17.1–42.6) and arginine (19.8 nmol/ml, normal range: 53.6–133.6) levels were decreased. Urinary orotic acid was extremely increased to 1735.7 mmol/molCr. These results confirmed the diagnosis of OTCD biochemically. Glucose was administered intravenously, and protein intake was restricted. Sodium benzoate, arginine, and citrulline were also administered. These treatments dramatically improved his clinical and biochemical abnormalities. After recovering from the first episode of hyperammonemia, he was treated with a protein-restricted diet (1.8 g/kg/day), sodium phenylbutyrate, and citrulline. Although he had four additional episodes of hyperammonemia due to infections or excessive protein intake, he developed and grew normally at the age of 3 years. His clinical course was consistent with a late-onset OTCD phenotype.

This study was approved by the Ethics Committee at Hyogo College of Medicine (Approval No. rinhi 113), and informed consent was obtained from the patient’s parents.

Genomic DNA from the peripheral blood was extracted using standard phenol-chloroform extraction methods, and genomic DNA from oral mucosa was extracted using the QlAamp DNA Mini Kit (Qiagen, Venlo, The Netherlands). A mutation analysis of 57 genes involved in urea cycle diseases was performed by multiplex PCR-based library preparation (1824 primers) and next-generation sequencing (NGS) (Miseq Sequencing System, Illumina, San Diego, CA, USA) at the Kazusa DNA Research Institute. Polymerase chain reaction (PCR) and direct sequencing analysis were also performed as previously described^15^.

To quantify the ratio between the normal and mutant alleles, a semiquantitative analysis was performed. gDNA was amplified by 27 cycles of PCR with a fluorescein-labeled reverse primer. Quantification was performed by measuring its area using GeneMapper software (Applied Biosystems by Thermo Fisher Scientific, Waltham, MA, USA) after PCR amplification.

NGS analysis and direct sequence analysis of genomic DNA from lymphocytes identified a mosaic frameshift mutation (c.834_840delCCAGGCT, p.Q279fs) in the *OTC* gene (Fig. 1a), revealing two different types of alleles, including a wild type and mutant with a frameshift mutation (c.834_840delCCAGGCT), in the patient. Only the wild-type allele was identified in the patient’s mother (Fig. 1a). His karyotype was 46, XY. These results suggested that a *de novo* somatic mosaicism caused OTCD. Because this mutation was a frameshift mutation, his clinical phenotype was expected to be a severe neonatal-onset phenotype. However, he exhibited a milder late-onset clinical course. This finding suggested that the wild-type allele could maintain OTC function despite the presence of a null mutation.

**Fig. 1:**
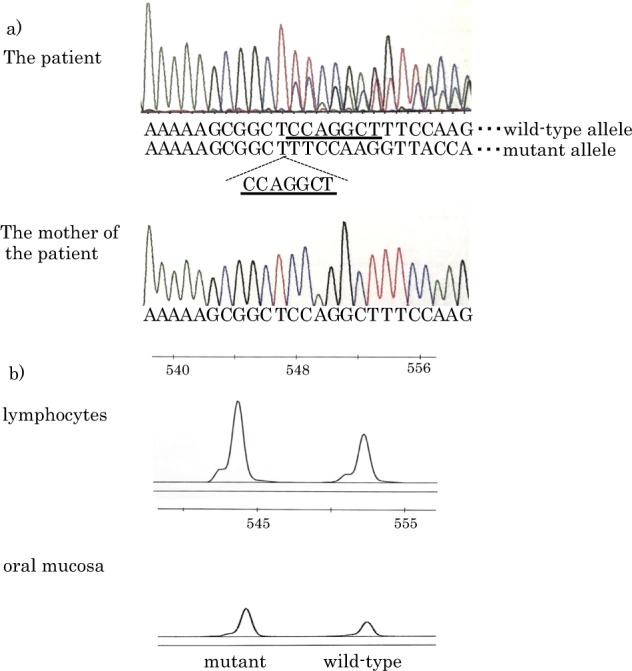
Nucleotide sequence and semiquantitative analysis of the *OTC* gene. **a** The results of a direct sequencing analysis of the *OTC* gene using genomic DNA from lymphocytes of the patient and his mother. Upper figure: Direct sequencing analysis revealed two different types of alleles: the wild-type and the mutant alleles with a frameshift mutation (c.834_840delCCAGGCT). Lower figure: Only the wild-type allele was identified in his mother. **b** Semiquantitative analysis using the GeneMapper software for lymphocytes and oral mucosa. The mutant allele was in 60.38 and 65.19% of the lymphocytes and oral mucosa cells, respectively

Next, we examined the mosaic ratio of the wild-type and mutant alleles. The mosaic ratio identified by NGS (62.73%, AmpliVar analysis) in the current case was doubtful due to a T homopolymer close to the mutation, which possibly caused misreading. Therefore, we performed semiquantitative analysis using GeneMapper software to determine the mosaic ratio in the lymphocytes and oral mucosa. The mosaic ratio was 60.38% in the lymphocytes and 65.19% in the oral mucosa (Fig. 1b). Unfortunately, other tissues were not investigated.

The mutation identified in the current patient was a novel mutation^16^. Despite the presence of a null mutation, this patient exhibited a milder late-onset phenotype due to mosaicism. The mutant allele was present in 60–65% of the lymphocytes and oral mucosa cells, which may vary among different tissues. Given that OTC is mainly expressed in the liver, the degree of mosaicism in the liver is interesting; however, this information was beyond the scope of the present study.

Postzygotic mutations with germline and somatic mosaics have been recognized as the cause of various genetic diseases^17^. In the patient in the present study, only somatic cells were investigated; therefore, somatic and gonosomal mosaicism can be considered.

To date, greater than 400 disease-causing mutations in the *OTC* gene have been reported^16^; however, somatic mosaic mutations have only been reported in only a few patients (Table 1). To the best of our knowledge, these six mutations have not been previously reported in male patients without somatic mosaicism. Only the mutation of patient #5 (L349P) was reported as female OTCD without detailed information^16^. Patients #1 and #2 exhibited milder late-onset phenotypes despite a large deletion mutation. Patient #4 and the current case also exhibited late-onset phenotypes despite a splice site and frameshift mutation, respectively. Despite the presence of a null mutation, these cases exhibited milder phenotypes, suggesting that the wild-type allele could rescue the function of OTC. For X-linked disorders in particular, such somatic mosaicism can explain mild forms of the disease in male patients^11^. Surprisingly, patients #3 and #5 with mosaic missense mutations exhibited no symptoms; however, their daughters exhibited severe phenotypes.

**Table 1:** Patients with OTCD caused by somatic mosaicism

Patient	Sex	Mutation in the *OTC* gene	Phenotype	Reference
#1	Male	exon 5–7 (8) deletion	Late	Maddalena et al.^11^
#2	Male	exon7–9 deletion	Late	Legius et al.^12^
#3	Male	c.444 G > C (p.L148F)	Asymptomatic	Komaki et al.^13^
#4	Male	c.386+1 G > T	Late	Qin et al.^14^
#5	Male	c.1046 T > C (p.L349P)	Asymptomatic	Qin et al.^14^
#6	Male	c.834_840delCCAGGCT (p.Q279fs)	Late	Current patient

Three daughters of patient #3 died during childhood due to hyperammonemia, and a heterozygous mutation (p.L148F) in the *OTC* gene was identified in the third daughter. Although it is unclear why all three daughters had severe symptomatic OTCD, biases for the inactivation of the maternal normal X chromosome apparently occurred^13^. The daughter of patient #5 experienced hyperammonemia, and a heterozygous mutation (p.L349P) was identified in the *OTC* gene. Unexpectedly, NGS analysis confirmed 21% mosaic for the same mutation in her father (patient #5) but not in her mother.

Interestingly, the daughters in these two families inherited mutations from the asymptomatic father with a mosaic mutation. Heterozygous female patients present a wide range of clinical manifestations due to random X-chromosome inactivation or the severity of the mutation^18^. In theory, heterozygous female patients exhibit milder phenotypes compared with male patients who are hemizygous for the same mutation. However, if the male patients have a mosaic mutation, this theory is not applicable. Therefore, female patients can have more severe phenotypes compared with male patients depending on skewed X-chromosome inactivation.

Therefore, for accurate genetic counseling, whether the mosaicism is also present in the gonads of this patient should be taken into consideration as a future daughter of this patient might become a carrier of severe OTCD. In particular, a future daughter of this patient can have a more severe phenotype than the daughters of patients #3 and #5.

These results also suggested that if *de novo* mutations in the *OTC* gene occur in females, efforts should be made to determine whether there is mosaicism in the father even if he appears to be asymptomatic. There can be cases with undetected mosaicism in the *OTC* gene. Determination of the presence of mosaicism is important for accurate recurrence-risk assessment and genetic counseling.

In summary, a somatic mosaic frameshift mutation was identified in a male patient with milder late-onset OTCD. Despite the presence of a null mutation, the current patient exhibited a milder phenotype. The presence of mosaicism can significantly impact the clinical phenotype and accurate recurrence-risk assessment, which should be taken into consideration for genetic counseling.

## Data Availability

The relevant data from this Data Report are hosted at the Human Genome Variation Database at 10.6084/m9.figshare.hgv.2363
